# A Decisive Metaheuristic Attribute Selector Enabled Combined Unsupervised-Supervised Model for Chronic Disease Risk Assessment

**DOI:** 10.1155/2022/8749353

**Published:** 2022-06-08

**Authors:** Sushruta Mishra, Hiren Kumar Thakkar, Priyanka Singh, Gajendra Sharma

**Affiliations:** ^1^School of Computer Engineering, Kalinga Institute of Industrial Technology, Deemed to be University, Bhubaneswar 751024, India; ^2^Marwadi University, Rajkot, Gujarat 360006, India; ^3^Department of Computer Science and Engineering, SRM University, Amaravati, Andhra Pradesh 522240, India; ^4^School of Engineering, Department of Computer Science and Engineering, Kathmandu University, Dhulikhel, Kavre 45200, Nepal

## Abstract

Advanced predictive analytics coupled with an effective attribute selection method plays a pivotal role in the precise assessment of chronic disorder risks in patients. Traditional attribute selection approaches suffer from premature convergence, high complexity, and computational cost. On the contrary, heuristic-based optimization to supervised methods minimizes the computational cost by eliminating outlier attributes. In this study, a novel buffer-enabled heuristic, a memory-based metaheuristic attribute selection (MMAS) model, is proposed, which performs a local neighborhood search for optimizing chronic disorders data. It is further filtered with unsupervised K-means clustering to remove outliers. The resultant data are input to the Naive Bayes classifier to determine chronic disease risks' presence. Heart disease, breast cancer, diabetes, and hepatitis are the datasets used in the research. Upon implementation of the model, a mean accuracy of 94.5% using MMAS was recorded and it dropped to 93.5% if clustering was not used. The average precision, recall, and F-score metric computed were 96.05%, 94.07%, and 95.06%, respectively. The model also has a least latency of 0.8 sec. Thus, it is demonstrated that chronic disease diagnosis can be significantly improved by heuristic-based attribute selection coupled with clustering followed by classification. It can be used to develop a decision support system to assist medical experts in the effective analysis of chronic diseases in a cost-effective manner.

## 1. Introduction

**Figure 1:**
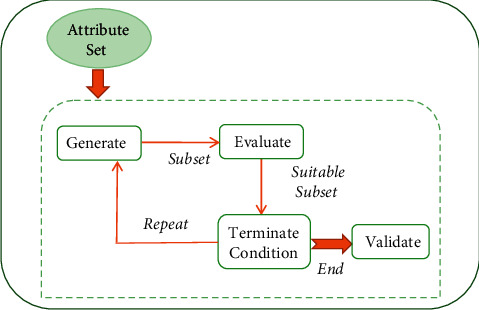
Attribute selection process.

According to healthcare data, the mortality rate of patients is quite high due to the rise in chronic diseases. The normal lifestyle of patients gets affected, and a lot of financial burden is also incurred by patients suffering from prolonged chronic disorders [1]. Thus, these disorders pose a great clinical challenge throughout the world. Hence, proper analysis of this issue at right time is crucial in order to minimize disease risks. With the constant growth of technology in the medical domain, recently data accumulation of patients is more convenient. Personal information, clinical history, and disease symptoms of patients are easily gathered in digital form in the form of electronic health record (EHR). These digital data of patients help in extracting relevant information about patients in real time with reduced cost and effort [2]. Meanwhile, the medical cost is rapidly increasing in comparison to the capability to deal with it. Moreover due to the availability of computers with technical assistance, a massive quantity of data is getting accumulated for processing. Decision-making of a human may be productive, but it is not up to the mark when the amount of data to be classified is massive and should be avoided in a sensitive real-time area like the clinical domain. Decision-making based on inconsistent clinical data records is a very common error observed during manual diagnosis [3]. Thus, it is better suited to enhance the usage of predictive learning models [4] in the medical field by implementing it as an intelligent problem-solving approach [5]. In the real world, heaps of data are regularly gathered and mainly in the healthcare industry processing these huge data are quite complicated. A chronic disease dataset may be comprised of numerous symptoms and attributes where not all of them are of equal importance in disease diagnosis [6]. Few of those attributes may be less relevant or may be noisy and redundant. The presence of these inconsistencies may degrade the overall performance of the predictive model and may create uneven delays in generating outcomes. Thus, a suitable attribute selection approach can be used to reduce the volume of such datasets but still maintaining the effectiveness of diagnosis. The suitable selection of an attribute selector is a challenging issue in predictive learning. In a disease sample set of “k” attributes, a total of “2k” subsets are feasible, among which the most optimum subset is to be chosen. In many scenarios, it becomes tough when the value of “k” is large since it may not be feasible to determine the model's performance for every subset of attribute [7]. Thus, attribute selection is applied to deal with such situations. In many previous works, several attribute selection methods like greedy search, exhaustive search, and random search are used to compute the best subset. But the majority of those methods are computationally expensive and complex along with untimely convergence [8]. In such cases, metaheuristic-based attribute selection methods are very efficient as they determine the best attribute subset, thereby maintaining the model's accuracy. Thus, metaheuristic-driven attribute optimization techniques can be implemented in optimizing chronic disease datasets to achieve an optimal efficiency in disease risk prediction, which can help in proper medical diagnosis.  Figure 1 illustrates a sample demonstration of the attribute selection procedure.

Besides metaheuristic methods, a suitable cluster analysis of the chronic disease datasets also can be utilized to segregate the attribute values exhibiting homogeneous traits, thereby recognizing the outliers. This research is based on developing and implementing an integrated hybrid unsupervised-supervised model for assessing chronic disease risks. A novel heuristic-based attribute selection method is utilized to eliminate less significant attributes from the data in quick time. The K-means clustering further identifies the outlier attributes, which is followed by classification with Naive Bayes. The result is quite promising and can be used as an assistive framework for clinical staff in the accurate and reliable diagnosis of chronic disorders in patients. The prime contributory elements of our research work are highlighted below:

- The impact of heuristic-based attribute selection on chronic disease datasets is studied.
- A novel memory-based heuristic attribute selection method (MMAS) is proposed to optimize the chronic disease datasets, which can be utilized for further classification using Naive Bayes.
- The attribute-optimized data are generated by integrating the heuristic MMAS method with the K-means clustering approach. Further, the output is subjected to classification to determine its efficiency using different parameters.
- Upon implementation, it is observed that the proposed model generated excellent outcome, and thus, it can assist medical experts in the effective and reliable diagnosis of chronic disorders.

## 2. Related Works

Common factors accountable for chronic disorders include age of patient, excess stress, heart risks, obesity, and less physical exercise. Many other symptoms are associated with different chronic diseases. All symptoms and risk factors are aggregated from digital datasets using an attribute selector. Then, it can be classified using suitable machine learning models. This section explores some existing and relevant attribute selection methods and predictive techniques used on chronic disease datasets to optimize the data samples. Simons et al. [9] used some predictive models like decision tables and neural networks for heart disorder data and it enabled them to predict Framingham risks in the heart for elderly people in Australia. Sah and Sheetalani [10] demonstrated the implementation of important predictive methods like nearest neighbors and support-vector machines to accurately predict cancer, liver, and heart risks from digital datasets. Patil et al. [11] deployed a computational analytics framework for the prediction of diabetes disease symptoms using clustering techniques, which was followed by decision tree classifiers.

It generated an impressive accuracy of 92.38%. Authors in [12] designed a predictive framework for the prediction of knee joint risks, and they used VAC signals for the purpose. Attribute selectors used were the apriori method and genetic search. The SVM was the classifier used. Piramuthu [13], applied many distance and probabilistic-based attribute selection techniques as a preprocessing method on chronic disease datasets. The outcome inferred that probabilistic measures are relatively less effective than interclass distance parameters. Karegowda et al. [14] proposed a novel categorization approach with the use of genetic search as wrapper and neural network for the classification of diabetic patients. The novel model proved to be better than neural network used alone. Authors [15] in utilized filter methods like Relief-F for attribute selection. Learning models like regression were applied for classification. Various evaluation measures like accuracy, precision, and recall were used. Relief-F with the SVM model outperformed others. Kolukisa et al. [15] used gain ratio and chi-square methods on the coronary artery dataset to detect less relevant features and remove them. Later, it was classified using random forest and it produced the best accuracy with least latency.

Hamsagayathri and Sampath [16] discussed the implementation of different decision tree algorithms on breast cancer data and concluded that the priority-driven decision tree gave the best performance with 93.63% accuracy. Kaur et al. [17] drew a comparative analysis of many attribute selection techniques and used evaluation metrics like kappa statistic, accuracy, positive rate, and latency to analyze the performance. Ramasamy et al. [18] applied decision tables, Hoeffding tree, logistic model tree (LMT), ensemble classifiers, and other trees to classify and compare risks of hepatitis. It was found that random forest recorded better performance than other algorithms. Hashem et al. [19], presented an integrated classifier approach that used alternating decision tree (ADT) and Pearson's correlation coefficient as attribute selector to predict liver fibrosis, thereby obtaining 84.8% accuracy.  Table 1 summarizes the overall important research works conducted using attribute selection techniques on chronic disease data.

An intelligent assessment model for kidney-related disorders is discussed in [34]. Different analytic methods like regression analysis, nearest neighbor, and decision tree were used for classification. Authors in [35] presented a predictive model to forecast various chronic risks using several machine learning methods like decision tree, ensemble classifiers, and probabilistic learners. A data mining-based disease recommendation system was developed in [36] that utilized online healthcare data records. A decision tree algorithm was used for improving the classification accuracy. Different immune and allergy symptom-based disease predictions were performed by authors in [37] using instance learners and margin learners. The main aim of the analysis was to determine the association between immunogens and chemical attributes of the datasets. A scalable chronic disorder risk detection system was developed in [38] using a random forest algorithm to deal with data skewing issues. A big data analytic-oriented approach for chronic disease assessment was deployed in [39] that involved different heterogeneous disease data samples. A decision tree was applied for classification, and MapReduce was used to enhance the operational efficiency. Authors in [40] applied neural network and ensemble learning methods for early and effective prediction of chronic kidney disorders.

## 3. Chronic Disease Dataset Details

Chronic disorder risks have become a significant concern throughout the world. In this study, four commonly detected chronic risk instances have been considered including diabetes, breast cancer, hepatitis, and heart disease data. They are perceived to be quite commonly spread chronic risks, and the digital data related to these diseases are available worldwide. The samples are retrieved and accumulated from the UCI repository.  Table 2 represents diabetes data sample information collected from the University of California. The PIMA Indian diabetes samples used in our study comprise eight unique attributes and 768 records. There are two class labels associated with it. The “0” indicates the absence of diabetes, and “1” denotes the presence of diabetes symptoms.

Table 3 represents the breast cancer samples utilized in the work. It is also collected from the UCI database. A cumulative ten attributes are available in the file exhibiting 2-class labels (recurrence or nonrecurrence).

The heart disease data, as depicted in Table 4, are also applied in the work. It constitutes 270 samples characterized by 2 distinct labels of class to determine whether any heart-related risks are found or not. The dataset has 13 different features.

Table 5 denotes the hepatitis dataset retrieved from the UCI repository. As observed, the dataset consists of 13 attributes and a class outcome that takes two values (either die or live).

The above four mentioned chronic disease datasets are applied in the research, upon which the proposed attribute selector is used to optimize the data. The reduced dataset is used for classification using Naive Bayes. The next section presents the proposed methodology model and its steps.

## 4. Proposed Methodology

The proposed model deals with designing a metaframework for chronic disease risk assessment by proposing a new heuristic-based attribute selector, thereby combining both supervised and unsupervised learning.

**Figure 2:**
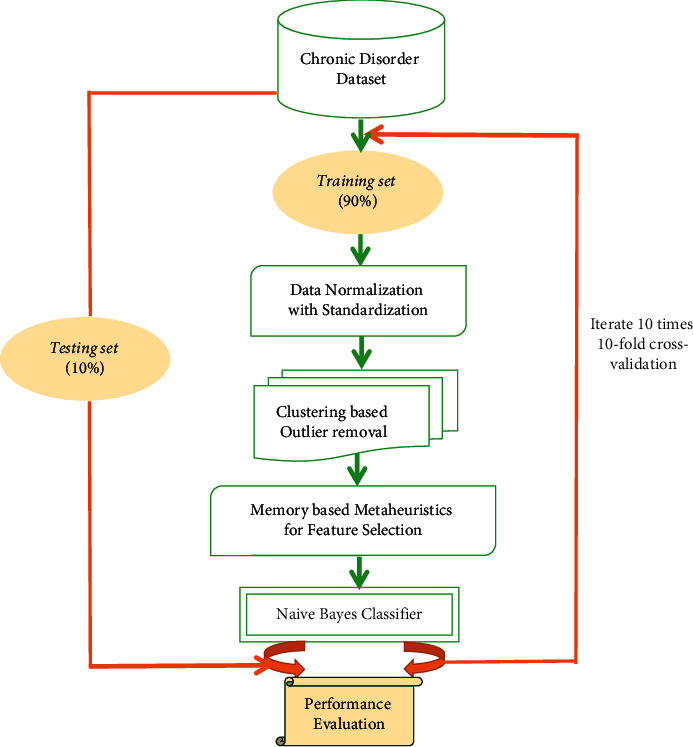
The proposed metaheuristic attribute selector-based classification model for chronic disorder detection.

Chronic disease datasets collected from the UCI repository are input to the proposed model as shown in Figure 2. The model depicts the use of the novel heuristic attribute selection method along with the Naive Bayes classifier. Mostly, the chronic risk data are unstructured, and so proper preprocessing and filtering are needed to map it in desired structure. So disease dataset preprocessing forms the next phase where any inconsistencies like comma, symbol, and delimiters are dropped by proper scanning of dataset. Other anomalies such as repeat values and missed out values are identified, which are replaced with the average value of the respective column. After successful preprocessing, min-max normalization is applied to the data to map all attribute values in homogeneous scaling. Here, each attribute is mapped to a decimal value range between 0 and 1. Equation (1) denotes the min-max normalization in the range of [0, 1] as follows:

(1) $$\begin{array}{c} p^{\prime }=\frac{p-\min\left(p\right)}{\max\left(p\right)-\min\left(p\right)}, \end{array}$$

where *p* denotes the original value, and *p*′ represents the normalized one. The feature reduced data are subjected to unsupervised learning using K-means for outlier detection. The K-means technique discloses data structure and generates clusters. At first, “n” features among the data *D* are chosen to initially form the center of the cluster. On the basis of the distance between cluster mean and attributes, identical objects are allotted to the cluster. For every cluster, the mean value is updated. The phase is repeated until there is no variation of features with an individual cluster. Here, a number of clusters need to be specified at prior. In the context of chronic risk analysis, two clusters are formed for the data samples. Equation (2) highlights the similarity between two attributes, which is computed through Euclidean distance, while equation (3) denotes the squared distance function between two vectors *a* = [*a*_1_, *a*_2_] and *b* = [*b*_1_, *b*_2_] as the summation of squared differences in coordinates. “dist” denotes the distance between the following:

(2) $$\begin{array}{c} dist_{a,b}^{2}={\left(a_{1}-b_{1}\right)}^{2}+{\left(a_{2}-b_{2}\right)}^{2}, \end{array}$$

(3) $$\begin{array}{c} dist_{a,b}=\sqrt{{\left(a_{1}-b_{1}\right)}^{2}+{\left(a_{2}-b_{2}\right)}^{2}}. \end{array}$$

After cluster formation is performed, the attributes not complying with any specific cluster are removed. The overall pseudocode for K-means clustering is depicted in Algorithm 1.

Memory-based metaheuristic attribute selection (MMAS) is the memory buffer-based heuristic method employing neighborhood search that is proposed and applied in this work. Here, a potential solution to a problem is traversed and its immediate nearest neighbors are looked upon so as to find a new better solution. The performance of the search is improved by accepting nonoptimal solutions if no more better solutions are explored. Also, already visited solutions are discouraged, which prevent any repetition of any solution space. The search implements a buffer structure to store the traversed solutions or rule set. If any solution is traversed at prior in a specific time duration or if any rule is violated, then that solution is no more considered. The pseudocode for the MMAS method is highlighted in Algorithm 2.

The pseudocode denotes preliminary setup, thereby building an initial solution selected at random. The initial solution is set as the optimum one at that instant and initializing a metalist with this initial solution. Here, a metalist is a memory buffer storage containing a set of elements of the stages traversed. The main iterative loop begins, and it continues searching for an optimum solution till the threshold fitness value as defined by the termination criteria is satisfied. The neighboring solutions are validated for the metalist elements. The algorithm tracks the optimal solution in the nearest solutions, which are not forbidden. The fitness function returns a score, which is considered as the new solution space is determined. If the newly found local solution exhibits a better fitness value compared to the present best, then it is considered to be the new best solution. The local best solution is included in the metalist, and if the metalist is full, then some elements will be permitted to expire. Usually, the elements expire from the list in the same sequence in which they are included. The process selects the best local solution so as to avoid the local optimum space. It further continues till the termination criteria are satisfied and at that instant the most optimum solution in the search is returned. The selected relevant attribute set is subjected to classification with the Naive Bayes classifier. This algorithm is a supervised method, which operates on the Bayes theorem. This classifier helps in prediction based on object probability. Bayes' theorem computes a hypothesis probability in context to prior knowledge as shown in equation (4).

(4) $$\begin{array}{c} p\left(\frac{x}{y}\right)=\frac{p\left(y/x\right)\times p\left(x\right)}{p\left(y\right)}. \end{array}$$

Here, *p*(*x*/*y*) denotes posterior probability, *p*(*y*/*x*) denotes likelihood probability, *p*(*x*) denotes prior probability, and *p*(*y*) denotes marginal probability.

**Figure 3:**
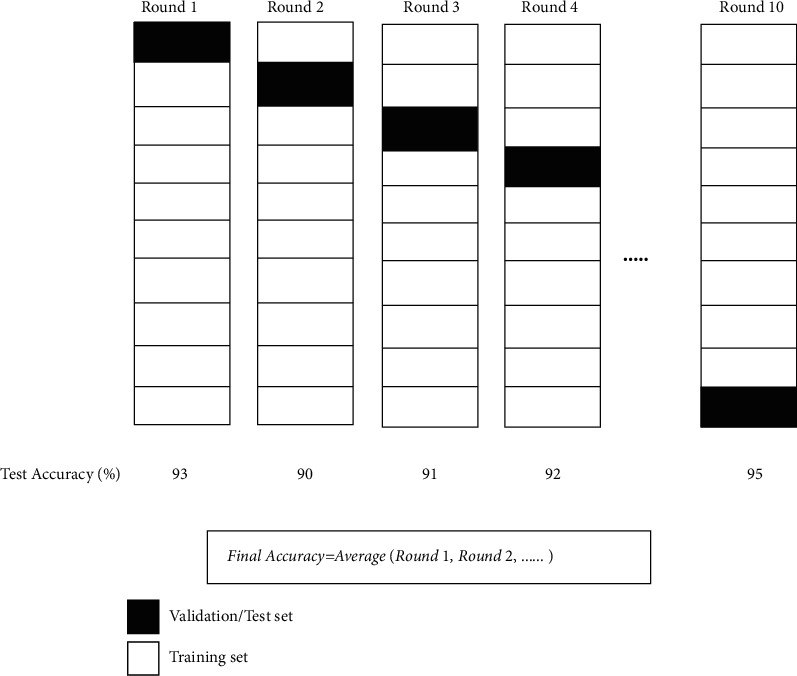
Demonstration of cross-validation method.

The defined attribute probabilities are determined. It is followed by the computation of the posterior probability using the Bayes theorem. The main objective of a prediction model is to ensure that the prediction is accurate upon test datasets provided. Thus, there is a need for a parameter that can determine the preciseness of a classifier when it is implemented on the testing dataset. The cross-validation technique is one such method that can solve this issue. Cross-validation is used to partition the entire dataset such that the training set section is large enough when compared to the validation set. The benefit of using cross-validation method is that it works well with all kinds of datasets and makes proper utilization of the entire dataset. Also, it prevents the model from overfitting and helps in fine-tuning the hyperparameters of the developed model. Then, this training set is used to train the system and use the test set to validate and compute our accuracy. In our research work, we have used a 10-fold cross-validation method. In this procedure, data are randomly sorted and then divided into 10-fold, and then, 10 iterations of cross-validation are run. In every iteration, one among the several folds is utilized to validate while the rest number of folds are to be used as training. Post-training of the classifier, its accuracy is computed on the validation set. The individual accuracy of all 10-fold is averaged to determine the final cross-validation accuracy, which is depicted in Figure 3.

## 5. Results and Analysis

The research discusses the impact of heuristic-based attribute optimization on optimizing the prediction performance of chronic disease risks by using a combination of unsupervised and supervised approaches. A new MMAS method of attribute selection optimizes the chronic disease datasets. The K-means clustering further eliminates outliers. Later, Naive Bayes classifies patients having chronic disorders. With color map related to ship encountering probability, the distribution of hot spots could be demonstrated for the sake of navigation safety. Also, the latency delay is very minimum and it can work well even with less data sample-based chronic disease datasets.

Different evaluation parameters were used to figure out the proposed model using confusion matrix values, which include true positives (TP), true negatives (TN), false positives (FP), and false negatives (FN). Classification accuracy is an important metric used to summarize the effectiveness of a classification framework as the correct prediction count divided by the total prediction count. It is shown in equation (5).

(5) $$\begin{array}{c} Accuracy=\frac{TP+TN}{TP+TN+FP+FN}. \end{array}$$

Precision determines the quantity of predictions of positive class, which genuinely belongs to the positive class as shown in equation (6).

(6) $$\begin{array}{c} \text{Precision}=\frac{TP}{TP+FP}. \end{array}$$

Recall represents the number of positive class predictions quantified of all positive instances in the data. Equation (7) shows the recall metrics.

(7) $$\begin{array}{c} \text{Recall}=\frac{TP}{TP+FN}. \end{array}$$

The F-score facilitates a single unified metric, thereby balancing both the issues of precision and recall in a single value, which is represented in equation (8).

(8) $$\begin{array}{c} F-score=\frac{2TP}{2TP+FP+FN}. \end{array}$$

Latency denotes the response time delay of the classification model in context to the time taken to generate the prediction outcome, which is highlighted in equation (9).

(9) $$\begin{array}{c} Latency=Model^{Train}+Model^{Test}. \end{array}$$

Table 6 highlights the impact of the use of the MMAS method on dataset attribute details of these chronic diseases along with the instance count of all datasets. When it is applied to heart disease data, the resultant attributes were reduced to 10. In the case of diabetes and breast cancer data samples, 2 less relevant attributes were dropped in each, while for hepatitis data, 15 important attributes were chosen after applying the MMAS method.

**Figure 4:**
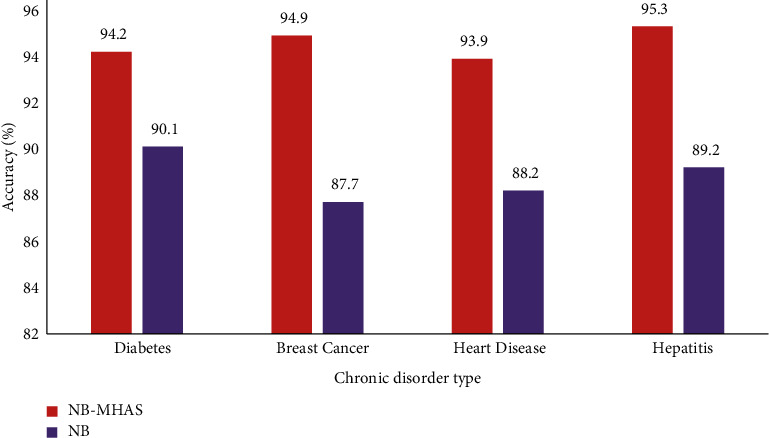
Classification accuracy analysis using the MMAS method on chronic disease data.

Figure 4 demonstrates the use of the proposed MMAS method on the classification accuracy of chronic disease datasets. As it is observed that in all four datasets, the classification accuracy is enhanced when the MMAS method is used rather than simply using the Naive Bayes classifier. Hepatitis data showed the best accuracy of 95.3% using the MMAS method. The mean accuracy obtained with MMAS is 94.5%, while 88.8% accuracy is noted if the Naive Bayes classifier is used without any attribute selector.

Performance of the new heuristic-based approach is analyzed with some existing popular attribute selection techniques like greedy stepwise (GSS), particle swarm optimization (PSO), and genetic search (GS) to determine its effectiveness, while Naive Bayes was the classifier used.

**Figure 5:**
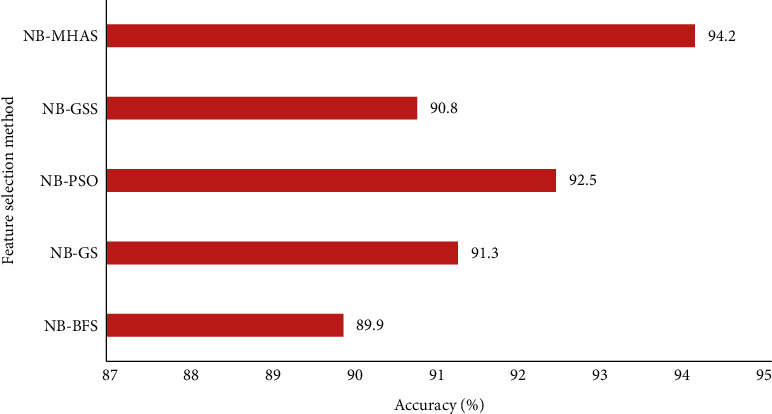
Comparison of the MMAS method with other heuristic methods for diabetes data.

The NB-MMAS method showed an excellent accuracy of 94.2% with diabetes data, while a relatively less accuracy of 89.9% was noted with the GSS method while NB was the classifier. The mean accuracy recorded with other heuristic methods is 91.1%.  Figure 5 shows the overall result of the comparative analysis of classification accuracy of the MMAS method with other heuristic approaches taking the diabetes dataset.

**Figure 6:**
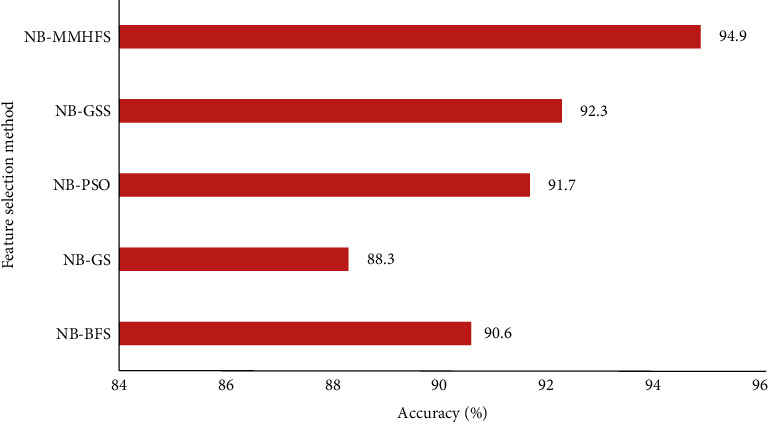
Comparison of the MMAS method with other heuristic methods for breast cancer data.

In the case of breast cancer data, NB-MMAS recorded an optimal accuracy of 94.9% while 88.3% accuracy was noted with the GS method using the same Naive Bayes classifier. The aggregated mean accuracy obtained with other heuristic methods was 90.7%. The result analysis of the implementation is shown in Figure 6.

**Figure 7:**
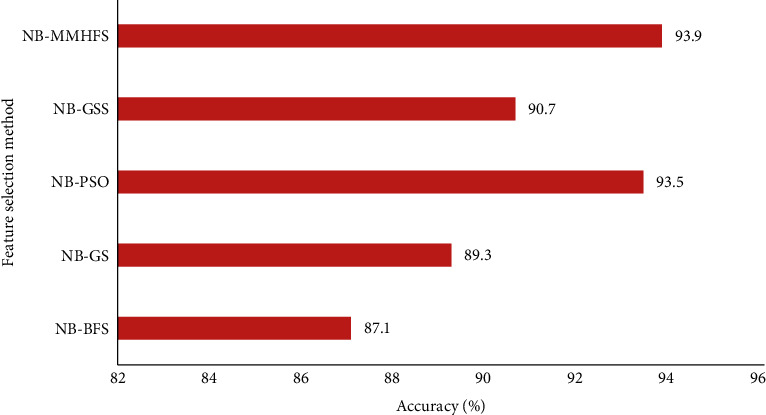
Comparison of the MMAS method with other heuristic methods for heart disease data.

In the case of heart disease data samples as shown in Figure 7, the NB-MMAS recorded an optimal accuracy of 93.9% while the NB-PSO also generated a very impressive accuracy of 93.5%. A comparatively low 87.1% accuracy was noted with the BFS method using the same Naive Bayes classifier. An average mean accuracy of 90.1% was noted with other methods, which is less than the accuracy obtained with the MMAS method.

**Figure 8:**
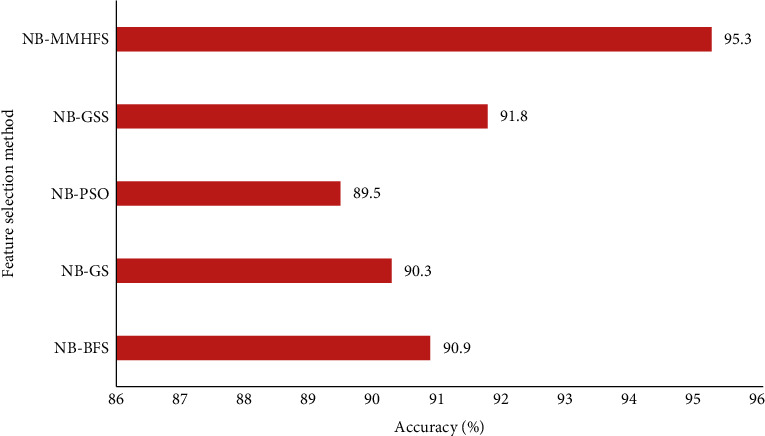
Comparison analysis of the MMAS method with other heuristic methods for hepatitis data.

Similarly, the NB-MMAS recorded an optimal accuracy of 95.3% and a less 89.5% accuracy with the NB-PSO method when subjected to the hepatitis dataset. A relatively less average accuracy of 90.6% was generated with other heuristic methods as depicted in Figure 8.

**Figure 9:**
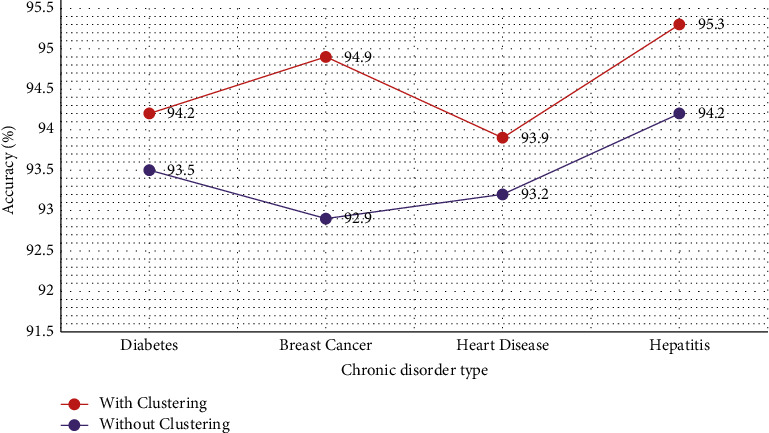
Impact of clustering on accuracy performance of the model.

The impact of clustering on the performance of chronic disease datasets was studied in Figure 9. It was noticed that clustering using K-means acted as a positive force, and it enhanced the accuracy of classification. A mean accuracy of 94.6% was noted when clustering was included, while a mean accuracy of 93.5% was the result without clustering.

The effectiveness of the MMAS method was evaluated with other learning indicators like precision, recall, and F-score. Both with and without using MMAS attribute selector were considered, and the outcome is highlighted in Table 7. The hepatitis dataset recorded the best precision, recall, and F-score values with 96.6%, 95.1%, and 95.85%, respectively. The average mean precision, recall, and F-score metric noted were 96.05%, 94.07%, and 95.06%, respectively.

**Figure 10:**
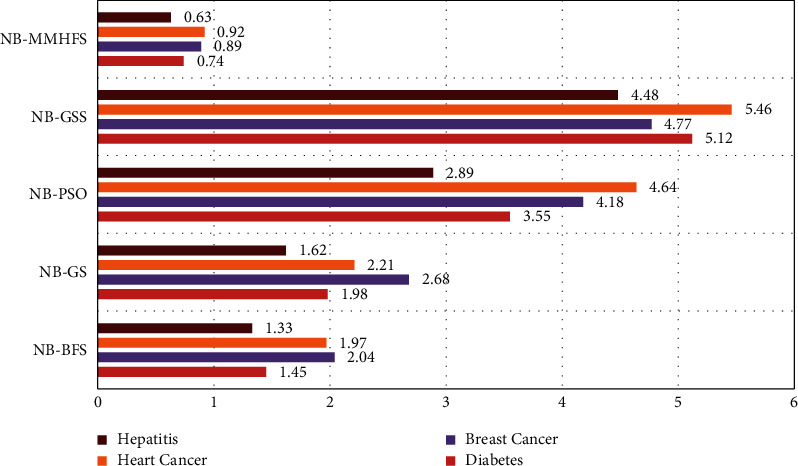
Latency delay comparative analysis using the MMAS method on chronic disease data.

The latency analysis was performed for different chronic disease datasets using various attribute selector-based classifications. Naive Bayes was the common classifier used in all cases. As depicted in Figure 10, it was observed that classification with the proposed MMAS method generated the best outcome. The latency period was found to be the least with the MMAS method on all datasets. The latency delay with hepatitis, heart disease, breast cancer, and diabetes disease data was recorded to be 0.63 sec, 0.92 sec, 0.89 sec, and 0.74 sec, respectively. Thus, a very less mean latency of 0.8 sec was computed using the MMAS method.

**Figure 11:**
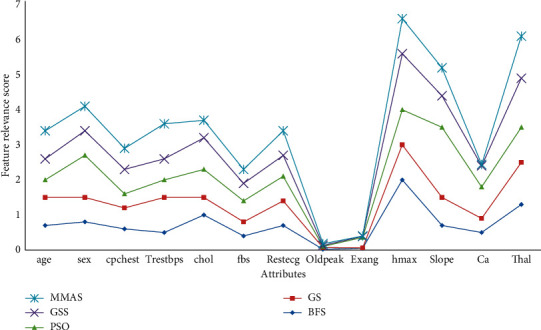
Feature relevance graph for heart disease dataset.

The importance of individual attributes in the chronic datasets taken into consideration upon applying heuristics is highlighted and compared with the other methods. This analysis is called feature relevance analysis. Here, the ranking of attributes on a score of 10 is graphically presented. Attributes are depicted on *X*-axis, and attribute score after applying heuristic methods is represented on *Y*-axis. On the basis of a low relevance score, those attributes are dropped from the result dataset. When the feature relevance analysis is conducted on heart disease data, it was observed that the attributes “oldpeak” and “Exang” computed the lowest relevance score with all heuristics as shown in Figure 11. Among all the methods, the MMAS recorded was able to generate the most optimal attribute set eliminating three attributes, which include “oldpeak,” “Exang,” and “ca”.

The overall result of feature relevance analysis on heart data is depicted in Table 8, where the MMAS records the most optimal dataset.

**Figure 12:**
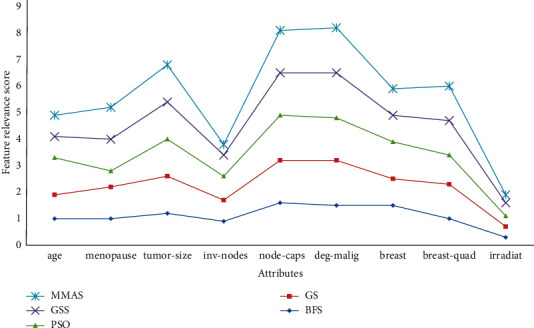
Feature relevance graph for breast cancer dataset.

The feature relevance analysis was also carried out on breast cancer data, and it was observed that almost all methods were able to successfully eliminate the least important attribute “irradiat” from the resultant set. Still, the MMAS method generated the best result dropping two less important attributes including “inv-nodes” and “irradiat” as shown in Figure 12.

Table 9 highlights the optimal outcome generated by the MMAS method as it can be seen that it is able to detect and drop two less relevant attributes from the final dataset, while other heuristics successfully detected only one less relevant attribute from breast cancer data.

**Figure 13:**
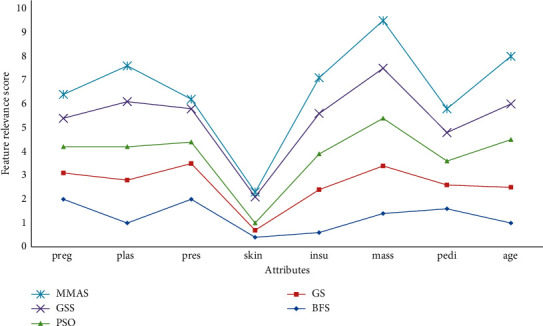
Feature relevance graph for diabetes dataset.

A detailed feature relevance analysis was undertaken on diabetes data as shown in Figure 13, and interestingly, almost all the heuristic methods failed to optimize the data samples except the MMAS method. While others were able to hardly identify one low relevance score attribute, the MMAS method computed the two least significant attributes, which include “skin” and “pres.”

As noted in Table 10, optimization with the MMAS heuristic method generated the best outcome with thereby identifying the two least attributes from the data samples of diabetes.

**Figure 14:**
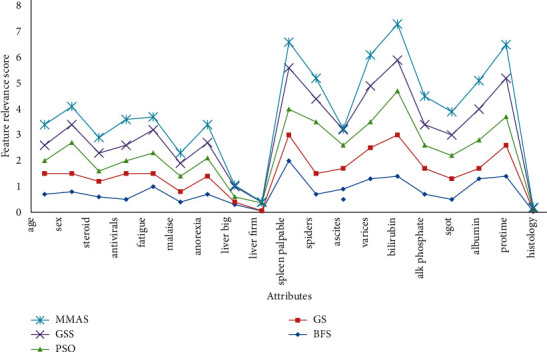
Feature relevance graph for hepatitis dataset.

When the feature relevance analysis is carried out on hepatitis data, not much impact is observed using heuristic methods as shown in Figure 14. With the majority methods, only two attributes were found to be least significant, while with the MMAS method, five attributes were detected as less relevant, and a more optimized attribute set is the output.

With the MMAS heuristic approach, as many as five attributes were reduced, which include “ascites,” “histology,” “malaise,” “liver firm,” and “liver big.” Thus, it generated a more optimum outcome as shown in Table 11.

**Figure 15:**
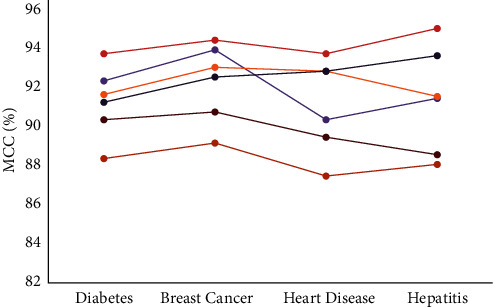
Matthews correlation coefficient (MCC) analysis.

**Figure 16:**
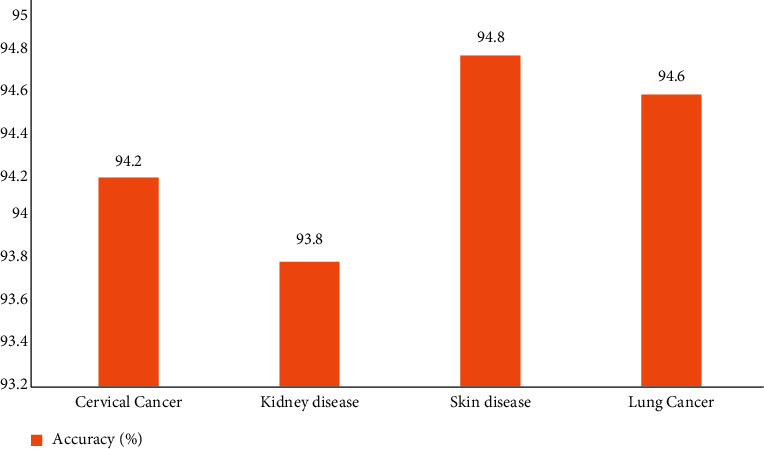
Accuracy analysis of the MMAS method on different disease datasets.

The Matthews correlation coefficient (MCC) is another evaluation parameter that can be used in machine learning-based classification. It determines the association of the true classes with that of the predicted classes. It computes a high score if the classification model accurately detected the majority of the positive data samples and negative data samples. The MCC metric was evaluated against the heuristic approach followed in this study on all chronic disease datasets as shown in Figure 15. The MMAS heuristic method determines the maximum MCC values in all four datasets as shown in Figure 16. While it generates 93.8% and 94.5% in diabetes and breast cancer data, it also records 93.8% and 95.1% values with heart disease and hepatitis datasets, respectively.

The proposed model can also be used in other risk disorder datasets. The authors have included a graphical analysis of the use of the proposed methodology in some other disease datasets.

The impact of the proposed MMAS method is evaluated using different datasets like cervical cancer, kidney disease, skin diseases, and lung cancer data. It is noted that the performance remains very consistent as it generates a very impressive accuracy rate with all these disease risks. The highest accuracy recorded was 94% with skin disease data samples.

## 6. Conclusion

Chronic disease symptom detection is a vital task in the medical field. This research analyzes the impact of attribute selection on chronic disorder instances. Breast cancer, diabetes, heart disease, and hepatitis are the datasets used in the study. The work deals with the proposal of a new heuristic-driven attribute selector, thereby developing an integrated metamodel that combines both supervised and unsupervised methods for chronic disease assessment. It presents a novel proposed heuristic-based attribute selector, the MMAS method that acts as an accurate attribute optimizer that picks the top relevant attributes of the chronic disease datasets. The K-means algorithm further drops the outlier attributes from the dataset. Later, Naive Bayes is used for the classification of patients' data to determine whether a patient has any major chronic disease symptoms or not. A mean accuracy of 94.5% was noted using the MMAS technique as compared to 88.8% accuracy when only Naive Bayes is used without any attribute selector. A mean accuracy of 94.6% was noted when clustering was included, while a mean accuracy of 93.5% was the result without clustering. The average mean precision, recall, and F-score metric noted were 96.05%, 94.07%, and 95.06%, respectively. Thus, a very less mean latency of 0.8 sec was computed using the MMAS method. Thus, the presented heuristic-based attribute selector was able to successfully optimize the chronic disease datasets, which were later used for the accurate detection of disease symptoms. The system model may be used to assist medical experts in the efficient diagnosis of chronic disease risks. In the future, the research study can be further enhanced to validate the model on more complex heterogeneous datasets with varying sizes and structures. Also, deep learning methods can be used using image-based real-time datasets.

## Figures and Tables

**Algorithm 1:**
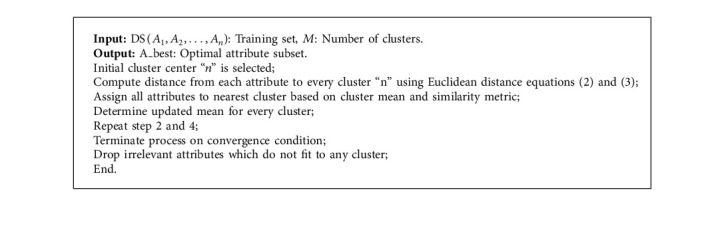
Cluster K-means.

**Algorithm 2:**
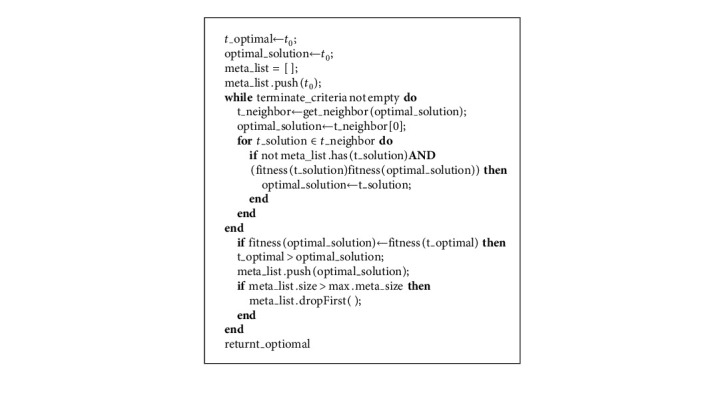
MMAS procedure.

**Table 1:** Existing work details on attribute selection over chronic disease datasets.

Existing works	Attribute selector used	Chronic disease dataset
El Akadi et al. [20]	Genetic algorithm	Dengue datasets
Mokeddem et al. [21]	Genetic algorithm	Coronary artery disease
Kora and Kalva [22]	Bat algorithm	ECG signal data
Keerthi Priya et al. [23]	Whale optimization algorithm	Breast cancer and hepatitis
Uzer et al. [24]	Artificial bee colony algorithm	Liver, diabetes, and hepatitis
Dogantekin et al. [25]	Linear discriminant analysis	Hepatitis datasets
Kohavi and John [26]	Sequential forward selection	Thyroid dataset
Gandhi and Prajapati [27]	Correlation feature selection	PIMA Indian diabetes
Kavitha and Kannan [28]	Principal component analysis	Heart disease dataset
Yildirim [29]	Consistency-based subset evaluation	Hepatitis dataset
Ding and Fu [30]	Information gain	Breast cancer and diabetes dataset
Kohli and Arora [31]	Adaptive boosting	Heart disease, breast cancer, and diabetes
Mishra et al. [32]	Genetic algorithm	Diabetes
Sahoo et al. [33]	DTNB algorithm	Heart disorders

**Table 2:** Diabetes dataset details [1].

Name of attribute	at-description	Domain range
Preg	Pregnancy count	0–15
Plas	Plasma glucose concentration	0–199
Pres	Diastolic blood pressure	0–122 (mm Hg)
Skin	Triceps' skin (mm) thickness	0–99 (mm)
Insu	Serum insulin (2-hour)	0–846 (mu U/ml)
Mass	Body mass index	0–67.1 (kg/m2)
Pedi	Diabetes pedigree function	0.08–2.42
Age	Person's age	21–81 years
Class	Label of person	0 = absence; 1 = presence

**Table 3:** Breast cancer dataset details [1].

Name of attribute	Description	Domain range
Class	Class label	Nonrecurrence and recurrence
Age	Age in years	10–19, 20–29, 30–39, 40–49, 50–59, 60–69, 70–79, 80–89, and 90–99
Menopause	Whether the patient is pre- or postmenopausal during treatment	ge40 or lt40 or premeno
Tumor-size	Tumour size (in mm)	0–4, 5–9, 10–14, 15–19, 20–24, 25–29, 30–34, 35–39, 40–44, 45–49, 50–54, and 55–59.
Iny-nodes	Total axillary lymph nodes that contain metastatic breast cancer	0–2, 3–5, 6–8, 9–11, 12–14, 15–17, 18–20, 21–23, 24–26, 27–29, 30–32, 33–35, and 36–39
Node-caps	If tumor penetrated in lymph node capsule	Yes or no
Deg-malig	Histological level of the tumor	1, 2, or 3
Breast	Which side of breast is affected	Right or left
Breast-quad	Breast is partitioned into four quadrants with nipple as the center	Right-up, left-up, right-low, left-low, and central
Irradiat	Patient's radiation (X-rays) therapy history	Yes or no

**Table 4:** Heart disease dataset details [1].

Name of attribute	Description	Domain range
Age	Age	1–100 years old
Sex	Person's gender	1 = male. 0 = female
Cp	Uneasiness in chest	General angina/nonanginal pain/asymptomatic/atypical angina/
Trestbps	Blood pressure at rest	Measured in mm Hg after admitted to medical centre
Chol	Serum cholesterol level	Measured in mg/dl
Restecg	Electrocardiogram outcome at rest time	Values of 0, 1, or 2
Oldpeak	Exercise-induced ST depression prior to rest	3.05–3.81
Exang	Exercise-induced angina	1 = yes; 0 = no
Smoke	Smoker or not	Value: 1 = yes; 0 = no
Slope	ST segment peak exercise slope	1: upsloping; 2: flat; 3: downsloping
Ca	Major vessel count	0–3
Thal	Maximum heart rate achieved	3 = normal; 6 = fixed defect; and 7 = reversible defect

**Table 5:** Hepatitis disease dataset details.

Parameters	Description
Class	Die, Live
Age	10, 20, 30, 40, 50, 60, 70, 80
Sex	Male, female
Steroid	No, yes
Antivirals	No, yes
Fatigue	No, yes
Malaise	No, yes
Anorexia	No, yes
Liver big	No, yes
Liver firm	No, yes
Spleen palpable	No, yes
Spiders	No, yes
Ascites	No, yes
Varices	No, yes

**Table 6:** Reduced dataset details after applying MHAS.

Chronic disease dataset	Dataset details	MMAS
Heart disease dataset	Samples	270
	Initial attributes	13
	Reduced attributes	10

Diabetes dataset	Samples	768
	Initial attributes	8
	Reduced attributes	6

Breast cancer dataset	Samples	286
	Initial attributes	9
	Reduced attributes	7

Hepatitis dataset	Samples	155
	Initial attributes	20
	Reduced attributes	15

**Table 7:** Impact of the MMAS method on different performance metrics.

	Diabetes	Breast cancer	Heart disease	Hepatitis
Without MMAS method				
Precision	90.8	89.9	89.9	90.4
Recall	90.2	85.6	87.6	88.1
F-score	90.5	87.7	88.7	89.2
With MMAS method				
Precision	95.5	96.3	95.8	96.6
Recall	94.4	94.1	92.7	95.1
F-score	94.9	95.2	94.2	95.8

**Table 8:** Impact of heuristics on heart disease dataset using feature relevance score.

Parameters	BFS	GS	PSO	GSS	MMAS
Number of instances	270	270	270	270	270
Initial attribute set	13	13	13	13	13
Reduced attribute set	11	11	12	11	10

**Table 9:** Impact of heuristics on breast cancer dataset using feature relevance score.

Parameters	BFS	GS	PSO	GSS	MMAS
Number of instances	286	286	286	286	286
Initial attribute set	9	9	9	9	9
Reduced attribute set	7	8	8	8	7

**Table 10:** Impact of heuristics on diabetes dataset using feature relevance score.

Parameters	BFS	GS	PSO	GSS	MMAS
Number of instances	768	768	768	768	768
Initial attribute set	8	8	8	8	8
Reduced attribute set	7	7	7	8	6

**Table 11:** Feature relevance graph for hepatitis dataset.

Parameters	BFS	GS	PSO	GSS	MMAS
Number of instances	155	155	155	155	155
Initial attribute set	20	20	20	20	20
Reduced attribute set	16	18	18	18	15

## Data Availability

The data are available at the UCI repository.
